# Case report: Single patellar metastasis of breast cancer

**DOI:** 10.3389/fendo.2025.1488216

**Published:** 2025-03-27

**Authors:** Xuefeng Ma, Yunyu Yan, Pengli Cai, Ying Liu, Ranxu Zhang, Yanhong Dong, Song Liu, Junfei Li, Jian Zhao, Ping Zhang

**Affiliations:** ^1^ Radiology Department, The 3rd Hospital of Hebei Medical University, Shijiazhuang, China; ^2^ Department of Orthopedic Surgery, The 3rd Hospital of Hebei Medical University, Shijiazhuang, China; ^3^ Hebei Orthopedic Research Institute, Key Laboratory of Biomechanics of Hebei Province, Shijiazhuang, China; ^4^ Radiology Department, Beijing Geriatric Hospital, Beijing, China

**Keywords:** breast cancer, patellar metastasis, CT, MRI, PET/CT

## Abstract

Breast cancer is recognized as the most prevalent malignant tumor among women and is prone to distant metastasis. However, patella metastasis in breast cancer cases is very rare. This case report presents the case of a 63-year-old Chinese female patient who experienced right knee pain and discomfort. The patient had a prior history of right breast tumor resection, and was diagnosed with single patellar metastasis of breast cancer.

## Introduction

The patella, the largest sesamoid bone in the body, is a rare site for tumors, whether primary or metastatic (1). While bone tumors frequently occur around the knee, primary tumors of the patella are uncommon, and patellar metastases are even rarer. Breast cancer metastasizing to the patella is exceptionally rare, accounting for only 2% of all patellar metastases (2).

According to the global estimates from the International Agency for Research on Cancer in 2020, breast cancer constituted 11.7% of all new cancer cases, making it the most common malignancy. Common sites of distant metastasis from primary breast cancer include the bone, liver, lungs, central nervous system, pleura, and peritoneum. Here, we present an unusual case of metastatic breast cancer only involving patellar metastasis. This case emphasizes the imaging manifestations of patients with a history of breast cancer when considering rare metastatic sites, as well as what kind of tests clinicians should order to further confirm suspicious diagnoses.

## Case presentation

**Figure 1:**
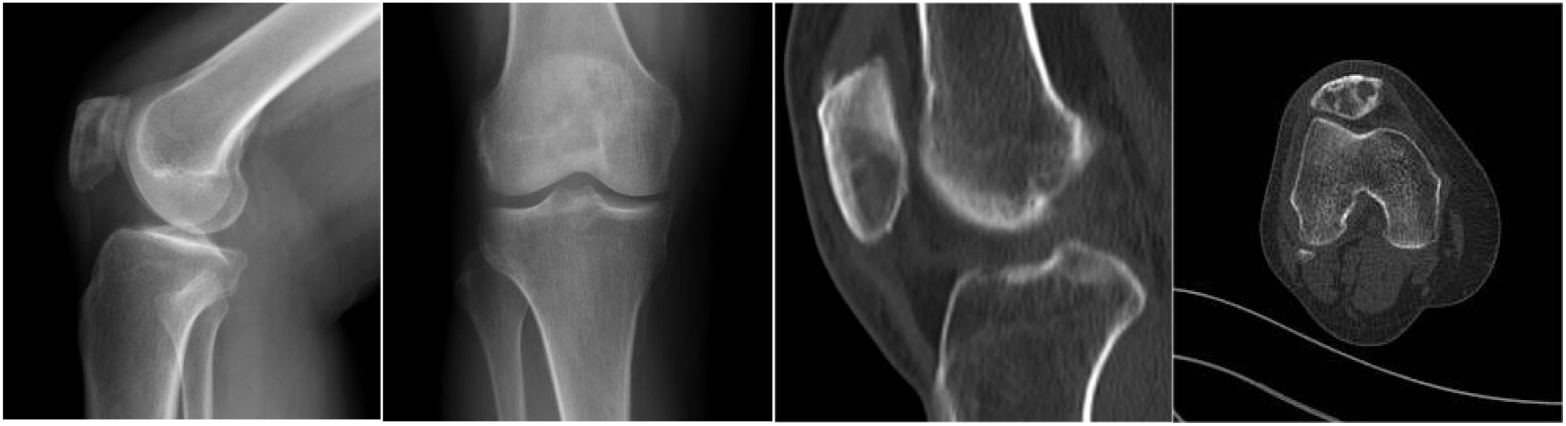
An X-ray examination showed an irregular low-density area on the right patella with clear edges. A computed tomography (CT) scan showed osteolytic bone destruction of the patella and unclear local cortex of the posterior margin.

**Figure 2:**
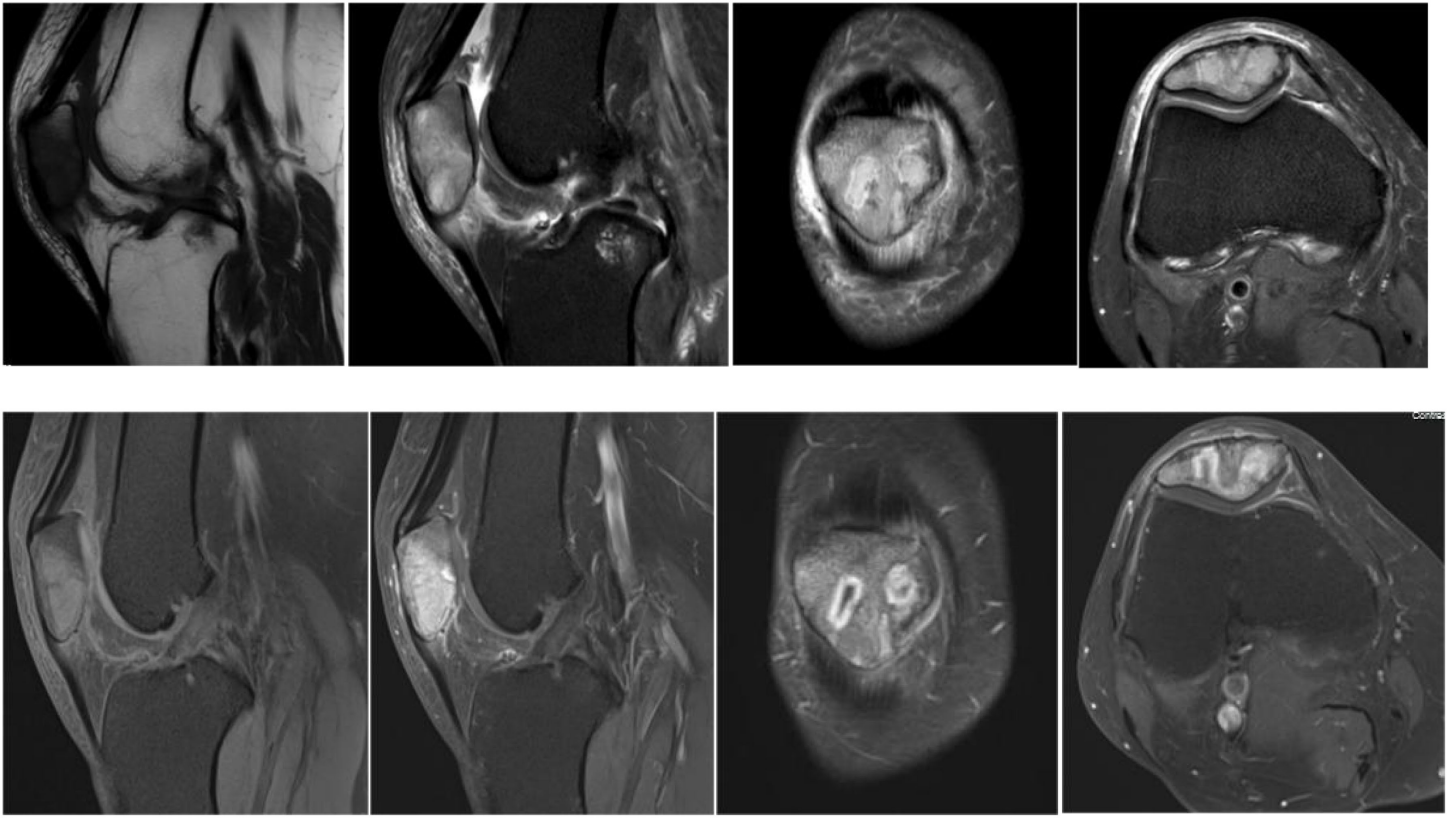
Magnetic resonance examination showed that irregular and slightly low signal on T1WI and slightly high and mixed abnormal signal shadow on T2WI could be seen in the medullary lumen of the right middle and lower patella, and low signal separation shadow could be seen in the lesion boundary. The articular cartilaginous surface of the posterior margin of patella was irregular, and the continuity of the medial posterior margin cortex was poor.

**Figure 3:**
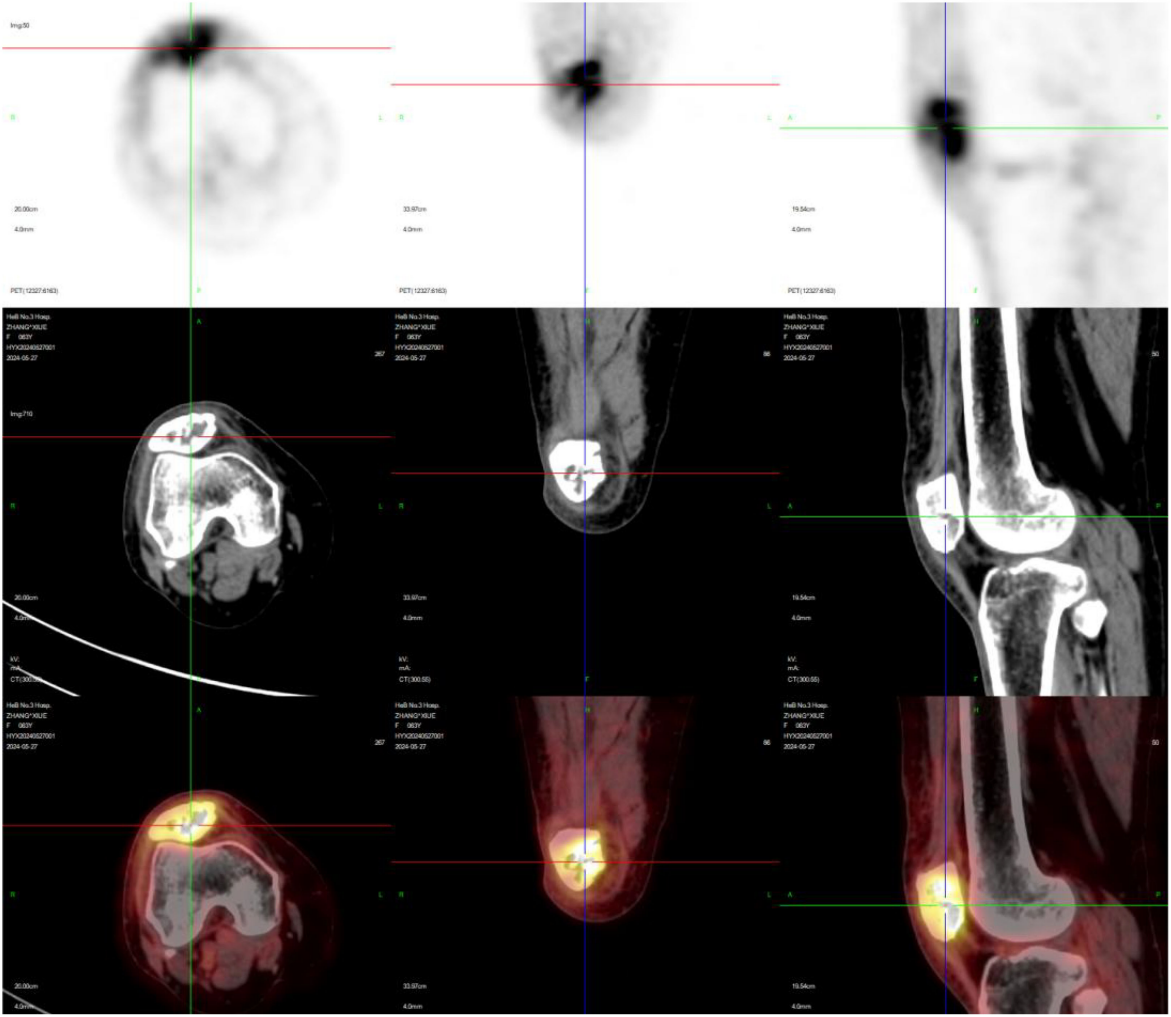
Further examination of positron emission tomography/computed tomography (PET/CT) revealed the local radiation uptake in the pulp cavity of the left upper femur was increased (SUVmax2.7). The bone morphology of the right patella was not regular, the bone was destroyed, and multiple flaky low-density shadows were seen, and the radioactive uptake was increased (SUVmax6.3). A nodule of increased radioactive uptake was seen at the right proximal tibia, SUVmax2.8.

**Figure 4:**
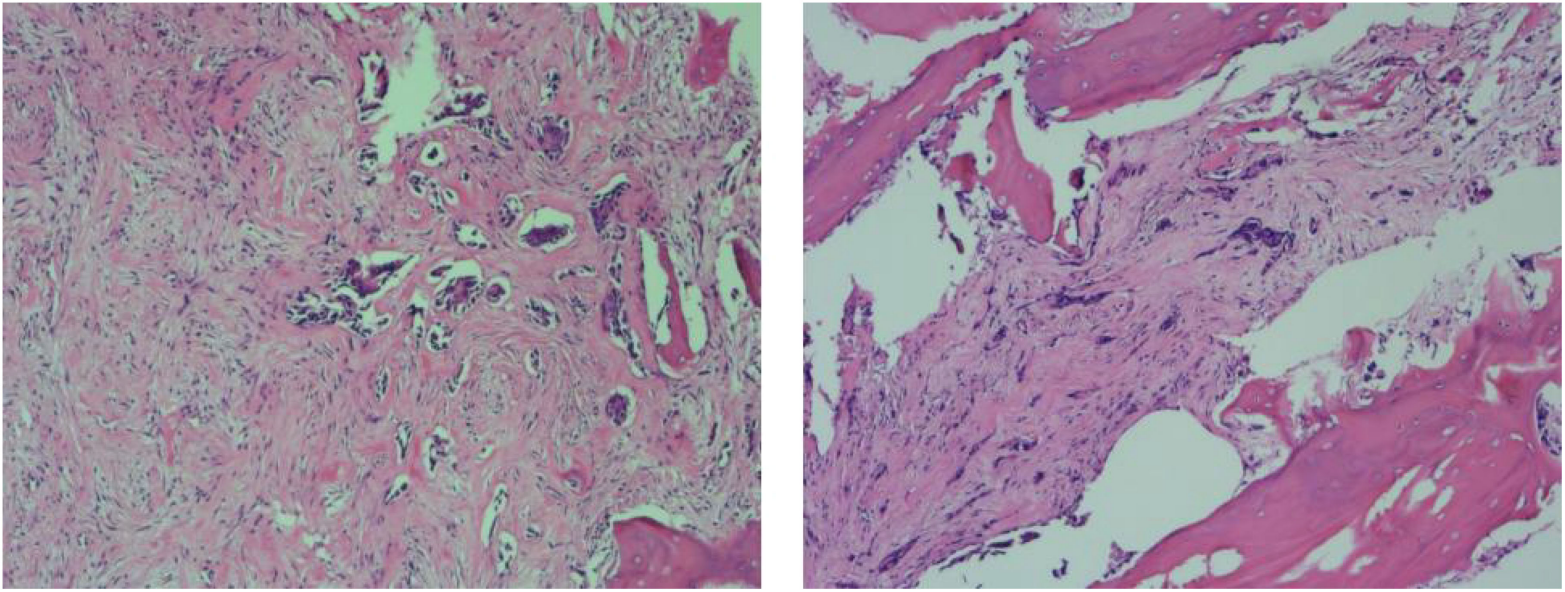
The patellar tumor was metastatic adenocarcinoma, consistent with the origin of the breast. The tumor cells strongly expressed ER (40%, strongly positive) and PR (60%, strongly positive), with Ki67 less than 5% of the tumor cells. When pathology, imaging results, and other assessments were analyzed in combination, the tumor was considered to be a breast cancer and metastasis to the patella.

The patient was a 63-year-old Chinese woman who experienced right knee pain and discomfort following an injury six months ago. She had been diagnosed with breast cancer nineteen years earlier. Following the surgery, she underwent radiotherapy and chemotherapy. Unfortunately, regular follow-up examinations were not maintained subsequently. On physical examination, the patient exhibited a limping gait and demonstrated tenderness upon gentle tapping of the right patella, although her knee movements were otherwise unremarkable restriction. Based on the patient’s complaints, medical history, and additional information, the clinician posed the question of potential metastatic foci in the patella and proceeded with a series of imaging examinations and clinical laboratory tests. All clinical laboratory tests yielded normal results. However, the imaging examinations revealed some abnormal findings. An X-ray examination revealed an irregular low-density region on the right patella, characterized by well-defined edges (**Figure 1**). A computed tomography (CT) scan indicated osteolytic bone destruction within the patella, accompanied by an indistinct posterior cortical margin (**Figure 2**). Magnetic resonance imaging (MRI) revealed irregular, moderately low signal intensity on T1-weighted imaging (T1WI), along with a combination of slightly elevated and abnormal signal intensities on T2-weighted imaging (T2WI) within the medullary cavity of the middle and lower patella. Additionally, low-signal separations were noted at the lesion boundaries, and the articular cartilage surface of the posterior patella exhibited irregularities, with disrupted continuity observed in the posterior medial cortical margin (**Figure 3**). Further examination using positron emission tomography/computed tomography (PET/CT) revealed increased radiotracer uptake in the medullary cavity of the left upper femur (SUVmax 2.7). The morphology of the right patella appeared irregular, with bone destruction and multiple areas of low density showing increased radiotracer uptake (SUVmax 6.3). A nodule with increased uptake (SUVmax 2.8) was also detected in the right proximal tibia (**Figure 4**). A needle biopsy, followed by a detailed pathological examination, confirmed that the patellar tumor was a metastatic adenocarcinoma, originating from breast cancer. Tumor cells showed strong expression of estrogen receptor (ER) (40%, strongly positive) and progesterone receptor (PR) (60%, strongly positive), with Ki-67 expression in less than 5% of the tumor cells. Based on the pathology, imaging and other assessments, the tumor was diagnosed as a breast cancer metastasis to the patella. Subsequently, the patient underwent curettage of the lesion in the right patella, followed by bone cement filling and radiotherapy as part of further treatment. The patient experienced a notable improvement in right knee pain, a reduction in skin temperature, and enhanced joint mobility compared to the pre-treatment state.

## Discussion

The patella is a sesamoid bone that originates from the quadriceps tendon, developing from cartilage precursor cells around the third month of gestation, and typically ossifying by the age of three (3). As the largest sesamoid bone in the human body, patellar bone tumors are rare, accounting for just 0.12% of primary bone tumors. Patellar metastases originating from breast tumors are even rarer, with an incidence of approximately 2% among all patellar metastases (4).

Most patellar bone tumors are benign, with giant cell tumors of bone, chondroblastomas, and aneurysmal bone cysts being the most common (3). Due to the patella’s nature as a sesamoid bone, lacking periosteum and having a poor blood supply, the likelihood of cancer cells metastasizing to the patella via blood circulation is relatively low. When metastases do occur, they often originate from primary tumors such as breast, prostate, lung, esophageal, cervical cancers, malignant melanoma, or lymphosarcoma (5).

X-ray imaging typically reveals osteolytic lesions (the most common feature), though osteoblastic and mixed bone patterns can also be observed. PET/CT is a highly sensitive method for detecting metastatic bone tumors. Reportedly, certain biomarkers can signal the potential for bone metastasis in breast cancer. These include dedicator of cytokinesis protein 4 (DOCK4), nuclear p21-activated kinase 4 (PAK4), peroxiredoxin-4 (PRDX4), L-plastin (LPC1), macrophage-capping protein (CapG), and GIPC interacting protein C terminus 1 (GIPC1), etc (6). Due to the rarity of patellar bone tumors, clinical presentations frequently remain asymptomatic or present merely as straightforward anterior knee pain. Insufficient familiarity with their imaging features can readily result in delayed diagnosis, thereby negatively impacting patient outcomes. Consequently, imaging studies are crucial for patients exhibiting unexplained anterior knee pain.

To ensure an accurate diagnosis, it’s often necessary to differentiate between common patellar tumors. (1) Giant Cell Tumor of Bone (GCTB), which primarily consists of mononuclear stromal cells and multinucleated giant cells. GCTBs are classified into benign and malignant forms and are most commonly found in individuals aged 20 to 40, with a slight predominance in women. These tumors are typically solitary and most frequently occur in the epiphysis of long bones and vertebral bodies, particularly the distal femur, proximal tibia, and proximal fibula. GCTBs originating in the patella are exceptionally rare. Typical X-ray features of GCTB include eccentric bone-end involvement, osteolytic and cystic destruction without periosteal reaction, lesion expansion, cortical thinning, and a soap-bubble appearance. Highly aggressive tumors may penetrate the cortex, leading to pathological fractures. Angiography often reveals a tumor rich in blood vessels, with arteriovenous fistula formation. (2) Aneurysmal Bone Cyst (ABC): This lesion is tumor-like in nature and is named for its resemblance to aneurysmal expansion due to the localized destructive process and the periosteal bone deposition surrounding the lesion. An ABC is a hemorrhagic bone cyst that expands from within the bone outward. It is filled with blood and contains connective tissue septa, which include fibroblasts, osteoclast-like giant cells, and reactive woven bone (7). ABCs are more common in adolescents, with the most frequent sites being the epiphysis of long bones, such as the proximal humerus and the spine. On X-rays, ABCs typically present as balloon-like, translucent, expansive, cystic osteolytic changes in the shaft or epiphysis of long bones. These lesions are often eccentric, well-demarcated, and separated by bony septa, which divide the cyst cavity into a honeycomb or foamy appearance. Occasionally, the lesion may also be centrally located. Microscopically, ABCs are characterized by blood-filled sinusoids of varying sizes and fibrous septa of varying thickness, with the blood sinuses appearing dilated and filled with blood (8). (3) Bone cyst: A bone cyst is a cystic, localized, tumor-like lesion that occurs within the medullary cavity, typically characterized by a single cavity filled with serous or serum-like fluid. It is most commonly seen in children and adolescents, with a predilection for the metaphyses of long bones such as the proximal humerus, proximal femur, proximal tibia, and distal radius. The majority of patients seek medical attention following a pathological fracture. X-rays typically reveal round or oval, well-defined osteolytic lesions in the metaphysis, often accompanied by varying degrees of cortical bone thinning and expansion. These lesions may be unilocular or multilocular and are frequently located adjacent to the epiphyseal growth plate, though they do not cross it. (4) Chondroblastoma: Chondroblastoma is a benign tumor arising from immature chondrocytes and is commonly found in the epiphysis or the ends of long tubular bones. It accounts for only 1% of all bone tumors, with the majority of cases occurring in individuals aged 10 to 20 years (9). On X-rays, chondroblastomas appear as oval lesions with thin sclerotic margins, located centrally or eccentrically in the epiphysis or bone apophysis. CT scans provide similar findings, showing periosteal reactions and cortical bone destruction. MRI typically reveals iso- to hypo-intense signals on T1WIand mixed signals on T2WI (10).

Common sites of distant metastasis from primary breast cancer include the bone, liver, lungs, central nervous system, pleura, and peritoneum. Andrew Nguyen et al. reported that breast cancer can also have leptomeningeal metastasis (11).But patellar metastases from breast cancer are rare. The treatment of single breast cancer with bone metastasis is usually surgical treatment, and the combination of chemotherapy reduces the risk of recurrence. New agents inhibiting PARP, CDK 4/6, PI3K, ILGF-1, estrogen pathways, HDAC, and HER2+ receptors and downstream effects, are being combined with traditional options such as radiation and surgery to develop new strategies to treat BCM (12). Some scholars also believe that some of the up-regulated genes such as Bcl3, Cxcl10, and Cxcr3; miRNAs (miR-497, miR-574, miR-138) and TFs (IRF8, STAT6, CCDN1) associated with metastasis may also be plausible targets for future therapeutics (13).

## Conclusions

There are few reports of metastatic adenocarcinoma of the patella in both domestic and international literature. For patients presenting with isolated anterior knee pain, particularly elderly individuals, it is crucial not to rely solely on clinical experience and mistakenly attribute the symptoms to degenerative osteoarthritis of the knee, which could delay proper diagnosis and treatment. Imaging and histopathological examinations are essential for accurately diagnosing patellar bone tumors. For patients suspected of having bone tumors, imaging examinations (X-ray, CT, MRI) are necessary, especially the diffusion-weighted imaging technology in MRI, which has great advantages in the diagnosis of benign and malignant tumors. For benign patellar tumors, thorough curettage, grinding, and either bone grafting or bone cement filling can be effective treatments. In cases of malignant patellar tumors, total patellectomy remains the most effective treatment option, and the combination of chemotherapy to reduce the risk of recurrence.

## Data Availability

The raw data supporting the conclusions of this article will be made available by the authors, without undue reservation.
